# A case of white bean allergy

**DOI:** 10.5415/apallergy.0000000000000111

**Published:** 2023-10-09

**Authors:** Teruaki Matsui, Nayu Sato, Masashi Nakamura, Yukiko Iwawaki, Katsumasa Kitamura, Yoshihiro Takasato, Shiro Sugiura, Kayoko Matsunaga, Komei Ito

**Affiliations:** 1 Department of Allergy, Allergy and Immunology Center, Aichi Children’s Health and Medical Center, Obu, Japan; 2 Department of Integrative Medical Science for Allergic Disease, Fujita Health University School of Medicine, Nagoya, Japan; 3 General Research and Development Institute, Hoyu Co., Ltd., Nagakute, Japan; 4 Comprehensive Pediatric Medicine, Nagoya University Graduate School of Medicine, Nagoya, Japan

**Keywords:** Down syndrome, Fabaceae, food hypersensitivity, white bean

## Abstract

White bean allergy is uncommon and rarely reported. Herein, we report a case of white bean allergy in a patient with Down syndrome. A 7-year-old girl with Down syndrome experienced allergic symptoms twice after eating white bean and visited our hospital for a food allergy investigation. An ImmunoCAP assay revealed a white bean-specific IgE (13.4 kU_A_/L) in the patient’s serum. In addition, her skin prick test result was positive. Moreover, ingestion of 2 g of boiled white beans in an oral food challenge test induced intermittent cough, desaturation, generalized urticaria, abnormal sleep, and mild hypotension. Thus, we diagnosed the patient with white bean allergy. We performed western blotting and mass spectrometric analysis and detected the following allergens: Phytohemagglutinin, group 3 late embryogenesis abundant protein, lipoxygenase, and legumin. In addition, we detected several candidate allergenic proteins for the first time. White bean, runner bean, or azuki bean was considered the primary source of sensitization because although immunoblotting inhibition tests revealed that the abovementioned beans inhibited other legumes, soybean, which she tolerates, showed little inhibition of the other legumes. However, we could not confirm whether the patient could ingest legumes other than soybean or white bean because her family did not wish to continue with further testing. This is the first report of a case of systemic allergic reactions to white bean in a child with Down syndrome. Further studies are needed to identify white bean allergens and understand the relationship between Down syndrome and white bean allergy.

## 1. Introduction

Soybean and peanut allergies are common; however, allergies to other legumes are relatively rare [1]. Legumes belong to the order Fabales, and the white bean (*Phaseolus vulgaris* L.) is a member of the *Fabaceae* family [2]. Although high rates of sensitization to white bean in the atopic population (51% in children and 39% in adults) have been reported in Morocco [3], white bean is usually well tolerated, even in children allergic to other legumes [4]. Moreover, there have been only a few reports of white bean allergy in France [5], Spain [6], and Japan [7]. Herein, we report a case of systemic allergic reactions to white bean in a patient with Down syndrome.

## 2. Case report

A 7-year-old girl with Down syndrome and a history of radical surgery for a ventricular septal defect, juvenile idiopathic arthritis, and suspected episodes of beta-lactam allergy visited our hospital for a food allergy investigation. At the age of 6, she ate white beans twice during lunch at school without any adverse reactions. At age 7, she experienced allergic symptoms twice after eating white bean soup. In the first episode, she had multiple hives, conjunctival hyperemia, and difficulty breathing 30 minutes after eating the soup. In the second episode, she vomited and had a swollen face and pale lips 15 minutes after eating the meal.

Informed consent was obtained from the patient’s guardians. This report was approved by the ethics committees of Aichi Children’s Health and Medical Center (2022009) and Fujita Health University (HM22-269).

We performed an ImmunoCAP assay (Thermo Fisher Scientific, Sweden) to measure the level of allergen-specific IgE in the patient’s serum. The results revealed a low total IgE (29 IU/mL) and a white bean-specific IgE (13.4 kU_A_/L). In addition, a skin prick test performed using boiled white bean returned positive results (Supplementary Table 1). Furthermore, ingestion of 2 g of boiled white bean in an oral food challenge (OFC) test induced intermittent cough, desaturation, generalized urticaria, abnormal sleep, and mild hypotension. Thus, the patient was diagnosed with white bean allergy.

**Figure 1.**
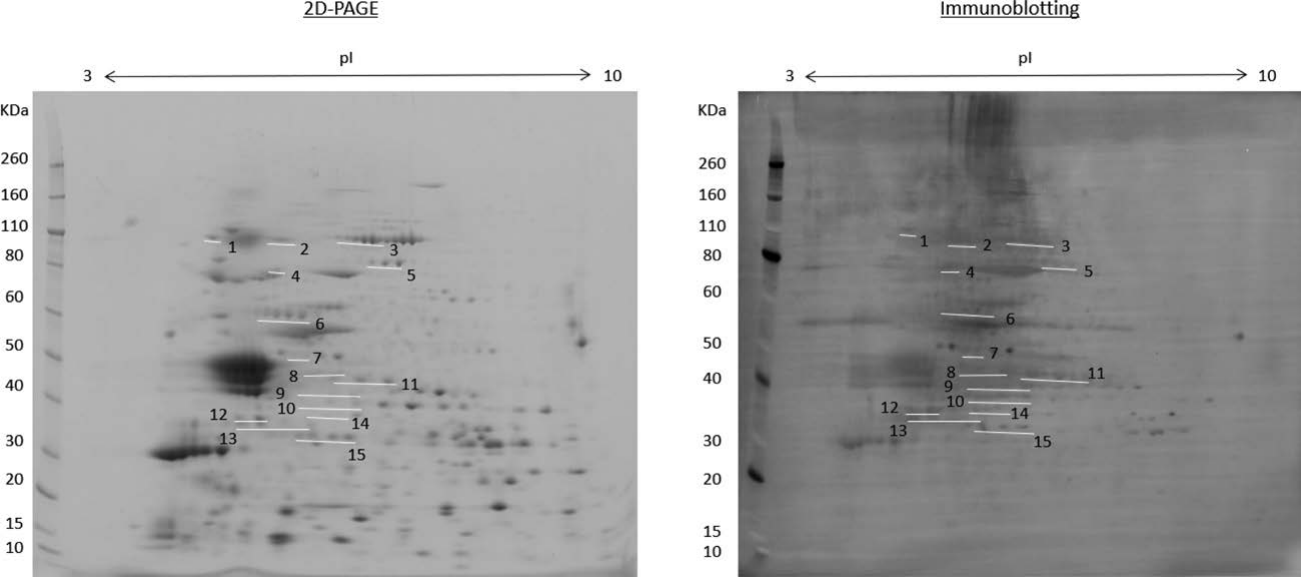
Two-dimensional electrophoresis and immunoblotting for the detection of white bean proteins. The image on the left depicts protein staining of white bean, whereas that on the right shows IgE detected through immunoblotting. Proteins for which IgE was specifically detected are underlined and numbered. These numbers correspond to the numbers in Table 1.

**Figure 2.**
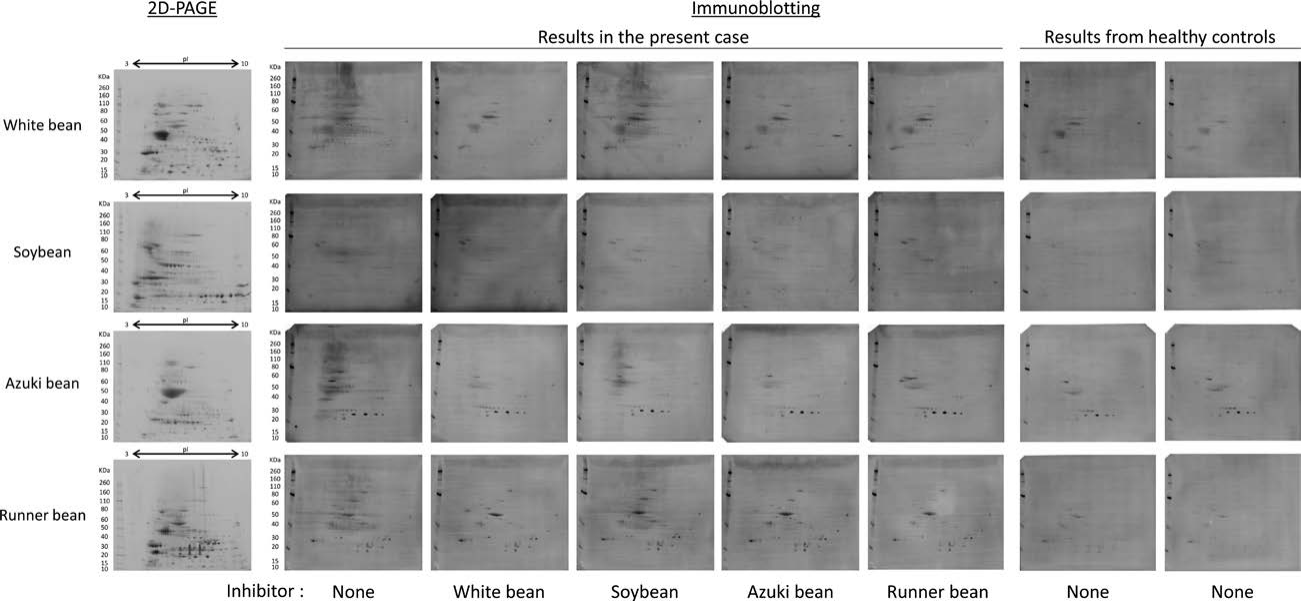
Two-dimensional immunoblotting and inhibition assay for each legume. Proteins from each legume were separated using two-dimensional electrophoresis, followed by immunoblotting, to confirm the binding of the IgE specific to this case. In addition, crossantigenicity was confirmed through inhibition assays of proteins from each legume. Healthy controls are volunteers with no legume allergies.

We performed western blotting (Figs. 1,2) and mass spectrometric analysis using the Universal Protein Resource database (UniProt) (https://www.uniprot.org) (Fabaceae) as previously described [7] to identify white bean proteins and antigens. In addition, we performed Basic Local Alignment Search Tool analysis using the National Center for Biotechnology Information database (https://www.ncbi.nlm.nih.gov) for assessment of the identified proteins that were not characterized in UniProt (Table 1). Phaseolin [5–7], Phytohemagglutinin [5], group 3 late embryogenesis abundant protein [7], lipoxygenase [7], and legumin [7] have been reported as suspected allergens so far. Although we did not detect phaseolin, which has been detected in several previously reported cases, we detected several allergens for the first time in the present case.

**Table 1.** Identification of white bean allergens using mass spectrometry

No.	Protein Pilot (Uniprot_Fabaceae)					Basic Local Alignment Search Tool			
	Accession	Name	Species	% Cov (95)	Peptides (95%)	Accession	Name	Species	Identities
1	V7CRB4	HATPase_c domain-containing protein	*P. vulgaris*	33.9	29				
2	V7AFT3	Poly [ADP-ribose] polymerase	*P. vulgaris*	36.5	21				
3	V7BZK0	Lipoxygenase	*P. vulgaris*	36.8	31				
4	V7CM38	Peptidase_M3 domain-containing protein	*P. vulgaris*	21.6	14				
5	V7CX68	5-methyltetrahydropteroyltriglutamate-	*P. vulgaris*	19.7	11				
		-homocysteine S-methyltransferase							
6	V7BJQ8	Uncharacterized protein	*P. vulgaris*	51.7	42	ABA26579.1	Group 3 late embryogenesis abundant protein	*P. vulgaris*	99%
7	D2DWA5	Formate dehydrogenase, mitochondrial	*P. vulgaris*	39.3	11				
8	V7BGE1	Uncharacterized protein	*P. vulgaris*	22.1	11	ADR30064.1	Legumin	*P. vulgaris*	96%
9	V7BGE1	Uncharacterized protein	*P. vulgaris*	21.6	11	ADR30064.1	Legumin	*P. vulgaris*	96%
10	V7BGE1	Uncharacterized protein	*P. vulgaris*	8.7	5	ADR30064.1	Legumin	*P. vulgaris*	96%
11	V7C8J3	Uncharacterized protein	*P. vulgaris*	41.7	26	XP_017413051.1	Late embryogenesis abundant protein D-29	*Vigna angularis*	73%
12	V7BC16	Lectin_legB domain-containing protein	*P. vulgaris*	47.3	12				
13	Q9M7M4	Mannose lectin FRIL (Fragment)	*P. vulgaris*	36.9	7				
14	V7BLB9	Malate dehydrogenase	*P. vulgaris*	28.6	9				
15	V7AIB2	Glyco_hydro_18 domain-containing protein	*P. vulgaris*	24.3	9				

% Cov (95): Percentage of the total sequence of the identified protein occupied by peptides detected with a confidence of >95%.

Peptides (95%): Number of peptides detected with a confidence of >95% using mass spectrometry.

Identities: Degree of identity with amino acid sequences of identified proteins registered in UniProt.

The protein from which IgE was specifically detected (Supplementary Fig. 1) was identified through mass spectrometric analysis performed using the Universal Protein Resource database (https://www.uniprot.org/) (Fabaceae) and a BLAST search performed using the National Center for Biotechnology Information database (https://www.ncbi.nlm.nih.gov).

*P. vulgaris*, *Phaseolus vulgaris.*

Crossantigenicity between white beans and red kidneyed beans [6], and among azuki beans, runner beans, and white pea beans [7] has been reported. In the present case, we performed skin prick tests to further investigate crossreactivity among legumes. All the tests returned positive results (Supplementary Table 1). In addition, the patient’s serum level of white bean-specific IgE was 13.4 kU_A_/L, whereas her serum levels of pea-, soybean-, and peanut-specific IgE were 1.38, 1.1, and 0.44 kU_A_/L, respectively. Immunoblotting inhibition tests revealed that white bean, azuki bean, and runner bean inhibited the other legumes. However, soybean showed little inhibition of the other legumes (Fig. 2), suggesting that 1 of the 3 abovementioned beans, rather than soybean, was the main sensitizer. Although a negative OFC test result confirmed that the patient tolerates soybean well, she did not want to undergo OFC tests for the other legumes.

## 3. Discussion

We present the first report of a case of systemic allergic reactions to white bean in a child with Down syndrome. In addition, we detected several allergens for the first time in the present case using western blotting and mass spectrometric analysis. Regarding crossantigenicity among legumes, the patient’s specific IgE levels and inhibition assay results suggested that white bean, runner bean, or azuki bean was the primary source of sensitization; however, we could not confirm whether she could ingest legumes other than soybean or white bean because her family did not wish to continue with further testing. Allergy to multiple legumes, including white, runner, and azuki beans, has been reported previously [7]; therefore, the patient in the present case may have been allergic to all the abovementioned beans.

Children with Down syndrome exhibit changes in their innate and adaptive immunity, which contribute to increased rates of autoimmune diseases, including juvenile idiopathic arthritis [8]. Schieve et al.[9] reported that parent-reported food allergies are common in children with Down syndrome. However, whether physician-diagnosed food allergies are prevalent among children with Down syndrome remains unclear. Food allergy complications in children with Down syndrome are rare in our routine clinical practice. Children with Down syndrome generally have low total IgE and show specific IgE sensitizations to common inhaled and food allergens less frequently than children without Down syndrome [8, 10]. In the present case, the patient’s total IgE level was low; however, her serum level of white bean-specific IgE was elevated, suggesting that her sensitization to legumes was a specific rather than overall susceptibility to allergens.

Further studies are required to identify the white bean allergens, evaluate the crossreactivity between white beans and other legumes, and understand the relationship between Down syndrome and white bean allergy.

## Conflicts of interest

KM is an endowed chair at Hoyu Co., Ltd. and received research funding from Hoyu Co., Ltd. NS and MN are employees of Hoyu Co., Ltd. The remaining authors declare no conflicts of interest.

## Author contributions

Teruaki Matsui, Nayu Sato, Masashi Nakamura, and Yukiko Iwawaki analyzed and interpreted the data and wrote the manuscript. Yukiko Iwawaki recruited the patient. Nayu Sato and Masashi Nakamura conducted the examinations. Masashi Nakamura, Kayoko Matsunaga, and Komei Ito supervised all the examinations and data analyses and confirmed the contents of the manuscript. All authors read and approved the final manuscript.

## Acknowledgements

We thank Editage (www.editage.com) for English language editing.
